# Hemodynamic monitoring strategies in cardiac surgery: an update systematic review

**DOI:** 10.1007/s10877-025-01407-7

**Published:** 2026-01-31

**Authors:** Rafael Melo, Vinicius Galindo, Luciana Gioli-Pereira, Daniel Joelsons, Murillo Assunção, Barbara Alves, Guilherme Souza, Bruno Bravim, Rogerio Passos

**Affiliations:** 1 Hospital Municipal Gilson de Cássia Marques de Carvalho, Hospital Israelita Albert Einstein, Av. Albert Einstein, 627/701, São Paulo, SP Brazil; 2 Department of Critical Care, Hospital Israelita Albert Einstein, São Paulo, SP Brazil; 3 Hospital Ortopédico Do Estado da Bahia, Hospital Israelita Albert Einstein, Salvador, BA Brazil

**Keywords:** Hemodynamic monitoring, Cardiac surgery, Goal-directed therapy, Perioperative care

## Abstract

Hemodynamic monitoring is a cornerstone of perioperative care in cardiac surgery, where patients are at high risk of cardiovascular instability and organ hypoperfusion. In recent years, goal-directed therapy (GDT) protocols have increasingly incorporated advanced monitoring technologies to optimize perfusion and improve outcomes. This systematic review aims to critically appraise contemporary hemodynamic monitoring strategies and their integration into GDT protocols in adult patients undergoing cardiac surgery. A systematic review of studies published between January 2015 and May 2025 was conducted using PubMed, Embase, Scopus, and the Cochrane Library. The last search was conducted on 17 May 2025 in all databases. Eligible studies included adult cardiac surgical patients managed with perioperative hemodynamic monitoring strategies that incorporated cardiac output assessment and structured GDT protocols. A qualitative synthesis of monitoring modalities, targeted hemodynamic endpoints, and reported clinical outcomes was performed. Our analysis included 15 studies comprising 4,224 patients. Monitoring strategies ranged from pulmonary artery catheters to minimally invasive and noninvasive tools such as FloTrac/EV1000 and esophageal Doppler. Cardiac index and stroke volume variation were the most frequently targeted parameters, often in combination with perfusion markers such as mean arterial pressure or central venous oxygen saturation. GDT protocols were associated with reductions in AKI, duration of mechanical ventilation, and ICU/hospital stay. Mortality benefits were inconsistently reported and not predefined in most studies. Current evidence supports the physiological rationale for GDT guided by advanced hemodynamic monitoring in cardiac surgery. Nonetheless, substantial heterogeneity in strategies and outcomes highlights the need for standardized protocols and high-quality multicenter trials to determine the most effective, patient-centered approaches.

**Trial registration:** PROSPERO registration number: CRD420251102582, retrospectively registered on 11 July 2025.

**Supplementary Information:**

The online version contains supplementary material available at 10.1007/s10877-025-01407-7.

## Introduction

Hemodynamic monitoring plays a pivotal role in the perioperative management of patients undergoing cardiac surgery, given the frequent occurrence of complex cardiovascular dysfunctions and the high risk of significant hemodynamic instability. The optimization of parameters such as cardiac output, tissue perfusion, and volume status is directly associated with a reduction in morbidity and mortality, underscoring the necessity for accurate monitoring strategies to guide therapeutic interventions [1].

Historically, invasive methods such as the pulmonary artery catheter (PAC) have been predominant in hemodynamic monitoring in cardiac surgery, particularly following the introduction of the Swan-Ganz catheter in the 1970s [2]. Although it revolutionized practice by allowing direct measurement of intracardiac pressures and cardiac output (CO), the use of the PAC has declined over time due to its associated complications and the lack of robust evidence demonstrating improvement in clinical outcomes [3].

Nevertheless, technological advancements have led to the development of minimally invasive and non-invasive hemodynamic monitoring modalities, including transpulmonary thermodilution (TPTD) [4], transesophageal echocardiography (TEE) [5], and pulse contour analysis (PCA) [6]. These tools provide cardiac index (CI) and several other hemodynamic parameters, with applicability variability depending on accuracy, patient characteristics, and operator expertise. For example, TEE has become indispensable intraoperatively due to its capacity for dynamic assessment of cardiac function and intra-vascular volume status [7].

Despite the wide array of available methods, considerable controversy persists regarding the optimal hemodynamic monitoring strategy in cardiac surgery, particularly in high-risk procedures. Factors such as the type of surgery, patient-specific clinical characteristics, and resource availability may critically influence the choice of monitoring modality. Moreover, incorporating emerging technologies, including artificial intelligence-based algorithms, has expanded the spectrum of clinical options [4]. However, the evidence remains heterogeneous and controversial, prompting us to undertake this systematic literature review to update current knowledge and inform evidence-based recommendations.

## Materials and methods

This review was retrospectively registered with the International Prospective Register of Systematic Reviews (PROSPERO) under registration number CRD420251102582 on 11 July 2025, and was conducted in accordance with the Cochrane Collaboration guidelines and Preferred Reporting Items for Systematic Reviews and Meta-Analyses (PRISMA) statement [8, 9]. PRISMA checklists are presented in the Supplementary Material. This study involved secondary data from previously published studies, exempting it from institutional review board approval. This review was conducted to provide a comprehensive and updated synthesis of the literature on hemodynamic monitoring strategies in adult patients undergoing cardiac surgery during the perioperative period. The search aimed to identify studies published in English between January 2015 and May 2025.

A systematic search was independently performed by two reviewers (R.H.M. and L.GP.) using four electronic databases (PubMed, Scopus, Embase, and the Cochrane Library). Disagreements were resolved through discussion with a third reviewer (V.G.). The last search was conducted on 17 May 2025 in all databases. The search strategy included a combination of keywords such as “cardiac surgery,” “heart surgery,” “coronary artery bypass grafting,” “hemodynamic monitoring,” and “goal-directed therapy”, combined with appropriate Boolean operators. Additional relevant studies were identified through citation tracking and backward reference searching of key articles and reviews.

Rayyan.ai software [10], a continuously updated, web-based platform for systematic reviews was used for screening and duplicate removal. After removing duplicates, titles and abstracts were screened, followed by full-text review to determine study eligibility. The final selection of studies was achieved through consensus among the three reviewers. Studies were included if they met the following criteria: (1) patients of age > 18 undergoing cardiac surgery; (2) hemodynamic strategies with cardiac output measurement described; (3) perioperative period; (4) incorporation of a goal-directed therapy (GDT) protocol. Exclusion criteria included preclinical studies, studies involving pediatric populations, case reports, conference abstracts, opinion articles, and editorials.

Data extraction was performed to capture essential study characteristics, including study design, type of cardiac surgery procedure, impact on clinical outcomes, and the hemodynamic monitoring tools employed. The objective was to conduct a qualitative assessment rather than a meta-analysis.

### Quality assessment

Two reviewers independently assessed the risk of bias for each study using the Cochrane Risk of Bias tool for randomized trials (Rob 2) for randomized studies according to the following domains: random sequence generation, allocation concealment, blinding of participants and personnel, blinding of outcome assessment, incomplete outcome data, selective outcome reporting, and other biases which categorizes studies as having a low risk of bias, some concerns, or a high risk of bias. For observational studies, the Risk Of Bias In Non-randomised Studies of Interventions (ROBINS-I) tool was applied.

## Results

**Fig. 1:**
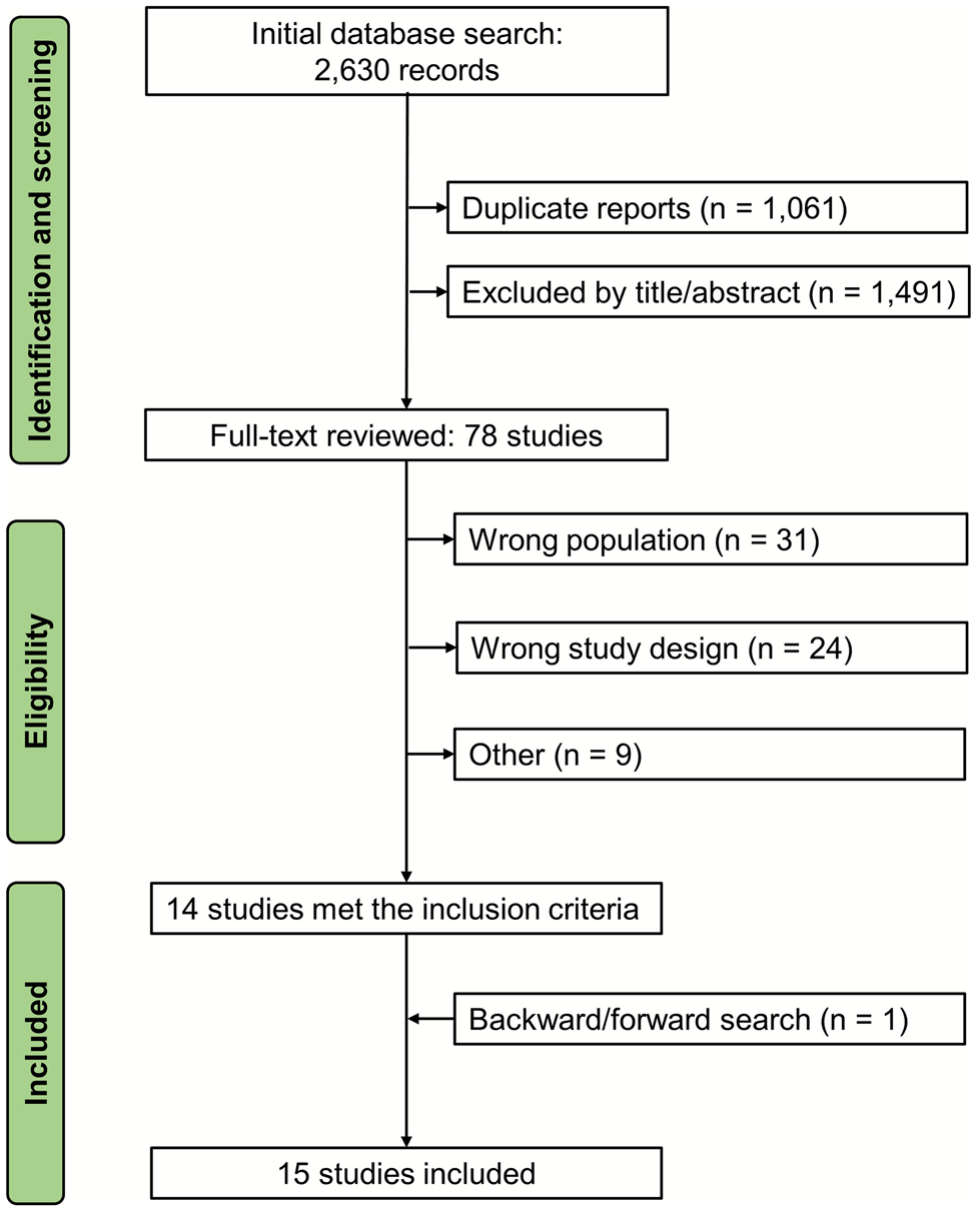
PRISMA flow diagram of search strategy and study selection

As shown in Fig. 1, the initial database search yielded 2,630 records. After removal of duplicates and title/abstract screening, 78 studies were selected for full-text review. Of these, 14 met the inclusion criteria. One additional study was identified through backward and forward citation tracking, resulting in a total of 15 studies included in this systematic review, comprising 4,224 patients. Study characteristics are summarized in Table 1. Among the 15 studies included, eight were randomized controlled trials (RCTs), while the remaining seven were observational, comprising three prospective cohort studies and four retrospective cohort studies. Sample sizes ranged from 40 to 1,979 patients. The mean/median age of participants ranged from 55.5 to 69 years, and the proportion of male patients varied from 49.7% to 80.7%. Most studies included patients undergoing coronary artery bypass grafting (CABG), either alone or in combination with valve surgery. CABG with cardiopulmonary bypass (CPB) was the most represented surgical population, while off-pump CABG was specifically assessed in three studies [11–13]. Some studies included mixed surgical cohorts, incorporating valve-only and CABG combined with valve surgery. There was substantial variability across studies in both hemodynamic monitoring strategies and therapeutic targets, reflecting evolving clinical practices over the last 10 years. Devices used included PACs, minimally invasive platforms of PCA (e.g., FloTrac/EV1000, LiDCOrapid), and adjunctive tools such as Non-Invasive Cardiac Output Monitor (NICOM®), esophageal Doppler, and renal biomarkers. Studies targeted a range of physiological endpoints, including CO, stroke volume variation (SVV), mean arterial pressure (MAP), and oxygen delivery index (DO_2_I).

**Table 1:** Characteristics and main findings of included studies

Study	Study Design	N (patients)	Age (years)	Male sex (%)	Type of surgery	Monitoring method (device)	GDT goals	Main outcomes evaluated
[14]	RCT	60 (30 PAC/30 Vigileo)	PAC-group: 55.6 ± 5.2; FloTrac-group: 55.5 ± 5.2	Not reported	CABG	PAC vs Vigileo-FloTrac	PAC-group: PAOP 12–18 mmHg; CI ≥ 2,0 L/min/m^2^; SvO₂ > 60%; MAP 60–100 mmHg; Flotrac-group: SVV < 12%; CI ≥ 2.5 L/min/m^2^; MAP maintained within “clinical range” (not prespecified numerically);	FloTrac group received more fluids and had shorter postoperative ventilation time
[18]	Retrospective Cohort	232 (GDT vs standard)	Pre-GDT group: 59.3 ± 17.1; GDT group: 59.7 ± 11.1	Pre-GDT group: 51.1%; GDT group: 49.7%	CABG, Valve only, CABG + Valve	PAC	CVP 10–12 mmHg, CI ≥ 2.4 L/min/m^2^, Lactate < 4 mmol/l and SvO2 > 65 mmHg	Protocol group received more fluid, and had a shorter duration of mechanical ventilation
[17]	Retrospective Cohort	1979 (725 pre- QI/1254 post-QI)	69 (59–76) pre-QI; 68 (60–75) post-QI	71%	CABG only, valve only and CABG + Valve	PAC, pleth variability index, passive leg raising, bedside transthoracic echocardiogram	CI > 2.5 L/min/m^2^, MAP > 65 mm Hg	Reduced AKI incidence
[19]	RCT	120 (60 GDT/60 control)	GDT group 61.17 ± 5.09; Control group 61.3 ± 5.60	68.33%	CABG	FloTrac and continous ScVO2 using PreSep	CI 2.5–4.2 L/min/m^2^; stroke volume index 30–65 ml/beat/m^2^; SVRI 1500–2500 dynes/s/cm^−5^/m^2^; oxygen delivery index 450–600 ml/min/m^2^; continuous ScVO2 > 70%, and stroke volume variation < 10%	Shorter ICU and hospital stay, and shorter duration of ventilation
[11]	RCT	142 (66 GDT/76 control)	GDT group 61.27 ± 5.47; Control group 61.26 ± 5.45	80.68%	Off-pump CABG	FloTracTM, PreSepTM, and EV‐1000® monitoring panels	MAP > 90 mmHg; CVP > 6–8 mmHg; U/O > 1.0 mL/kg/h; SpO2 > 95%; Hct > 30; ScVO2 > 70%; CI > 2.5 L/min/m^2^; SVV < 10%; SVRI > 1500–2500 dynes/s/cm^−5^/m^2^; DO_2_I > 450–600 mL/min/m^2^; Stroke volume index > 30–65 mL/beat/m^2^; GEDV > 680–800 mL/m^2^; EVLW = 3–7 mL/kg, < 10 mL/kg	Lower ICU and Hospital LOS
[15]	RCT	58 (29 PAC/29 NICOM)	PAC group 60.2 ± 12.6; NICOM group 62.6 ± 11.4	75,86%	Valve only	PAC vs NICOM	MAP 60–80 mmHg, CI ≥ 2.0 L/min/m^2^	No difference in primary outcome (hospital LOS); Patients requiring epinephrine and ventilator care > 24 h higher in the PAC group. PAC required larger amounts of colloid and vasopressors
[28]	RCT	276 (138 intervention group/138 control group)	Control group 68.33 ± 11.6; Intervention group 68.4 ± 11.2	72.1%	CABG, Valve only,and CABG + Valve	Central venous pressure, arterial pressure, urine output, NephroCheck® ([TIMP-2]·[IGFBP7]); PiCCO	Implement KDIGO bundle: maintain MAP > 65 mmHg,	Reduced AKI frequency and severity after cardiac surgery in high-risk patients
[22]	RCT	126 (62 GDT/64 control)	66 ± 9	73%	CABG, Valve only, CABG + Valve	LiDCOrapid	CI > 3 L/min/m^2^	30-day mortality and major postoperative complications (primary outcome) reduced om GDT group; GDT group received more fluid, with no difference in inotropes or transfusions; GDT group had a lower incidence of infection and low cardiac output syndrome
[23]	RCT	60 (30 GDT/30 control)	62.5 ± 9.6	77%	CABG, Valve only, CABG + Valve	Esophageal Doppler (CardioQ-ODM +, Deltex, Chichester, UK)	SVI > 30 mL/beat/m^2^; Optmize MAP	Similar AKI in both groups; Decreased Cystatin-C levels; GDT fluid therapy decreased fluid and erythrocyte requirements with shorter length of hospital stay
[27]	Prospective Cohort	478 (GDT vs standard care)	56 ± 14	61.5%	CABG, Valve only, CABG + Valve	CVP, MAP, urine output, and ScvO₂	CVP 6–8 mmHg; MAP 65–90 mmHg; Urine output ≥ 0.5 mL/kg/h; ScvO₂ ≥ 70%	GDT reduced the postoperative ventilatory period, frequency of changes in inotropes, incidence of AKI
[20]	Prospective Cohort	550	Group 1 65.5 ± 15.25; Group 2 64 ± 14; Combined Group 1–2 65 ± 15; Group 3 62 ± 15.75	66.36%	CABG or Valve only	Flotrac/EV1000	Patient-specific MAP and CI targets	Reduced ICU stay and inotrope use
[21]	Retrospective Cohort	60 (30 EV1000/30 control)	EV1000 64.5 ± 8.1; Control 65.3 ± 7.5	66.67%	CABG	FloTrac/EV1000	SVV < 13%; CI of 2.2–4.0 L/min/m^2^; SVRI 1500–2500 dynes/s/cm^−5^/m^2^	Shorter ICU/hospital stay, cost-effective
[12]	RCT	40 (20 GDT/20 control)	GDT 68.1 ± 5.7; Control 65.1 ± 9.3	75%	Off-pump CABG	FloTrac/EV1000	SVV < 13%; CI of 2.2–4.0 L/min/m^2^; SVRI of 1500–2500 dynes/s/cm^−5^/m^2^	Reduced ICU and hospital stay
[16]	Prospective Cohort	80 (40 GDT/40 control)	Usual-care 67.7 (11.5); Study-protocol 64.4(13.7)	Usual-care 62.5%; Study-protocol 65%	CABG, Valve only, CABG + Valve	PAC	CI ≥ 2 L/min/m^2^, SvO₂ ≥ 60%, MAP 65–75 mmHg	No difference in duration of noradrenaline or mechanical ventilation; Higher reintubation rate in GDT group
[13]	Retrospective Cohort	131 (75 GDT/56 control)	Not reported	55.73%	Off-pump CABG	Vigileo/FloTrac EV1000 platform	SVV ≤ 12%; CI ≥ 2.5 L/min/m^2^; MAP ≥ 65 mmHg	Reduced complications and shorter ICU/hospital stay

AKI – Acute Kidney Injury, CABG – Coronary Artery Bypass Grafting, CI – Cardiac Index, CVP – Central Venous Pressure, DO₂I – Oxygen Delivery Index, EV1000 – Edwards Lifesciences Monitoring Platform, FloTrac – FloTrac Monitoring System, GDT – Goal-Directed Therapy, GEDV – Global End-Diastolic Volume, Hct – Hematocrit, ICU – Intensive Care Unit, LiDCOrapid – LiDCOrapid Monitor, LOS – Length of Stay, MAP – Mean Arterial Pressure, NICOM – Non-Invasive Cardiac Output Monitor, PAC – Pulmonary Artery Catheter, PAOP – Pulmonary Artery Occlusion Pressure, PiCCO – Pulse Contour Cardiac Output, RCT – Randomized Controlled Trial, ScvO₂ – Central Venous Oxygen Saturation, SpO₂ – Peripheral Oxygen Saturation, SVI – Stroke Volume Index, SVRI – Systemic Vascular Resistance Index, SVV – Stroke Volume Variation, SvO₂ – Mixed Venous Oxygen Saturation, TIMP-2 – Tissue Inhibitor of Metalloproteinases-2, IGFBP7 – Insulin-like Growth Factor Binding Protein 7, U/O – Urine Output

### Quality assessment

**Fig. 2:**
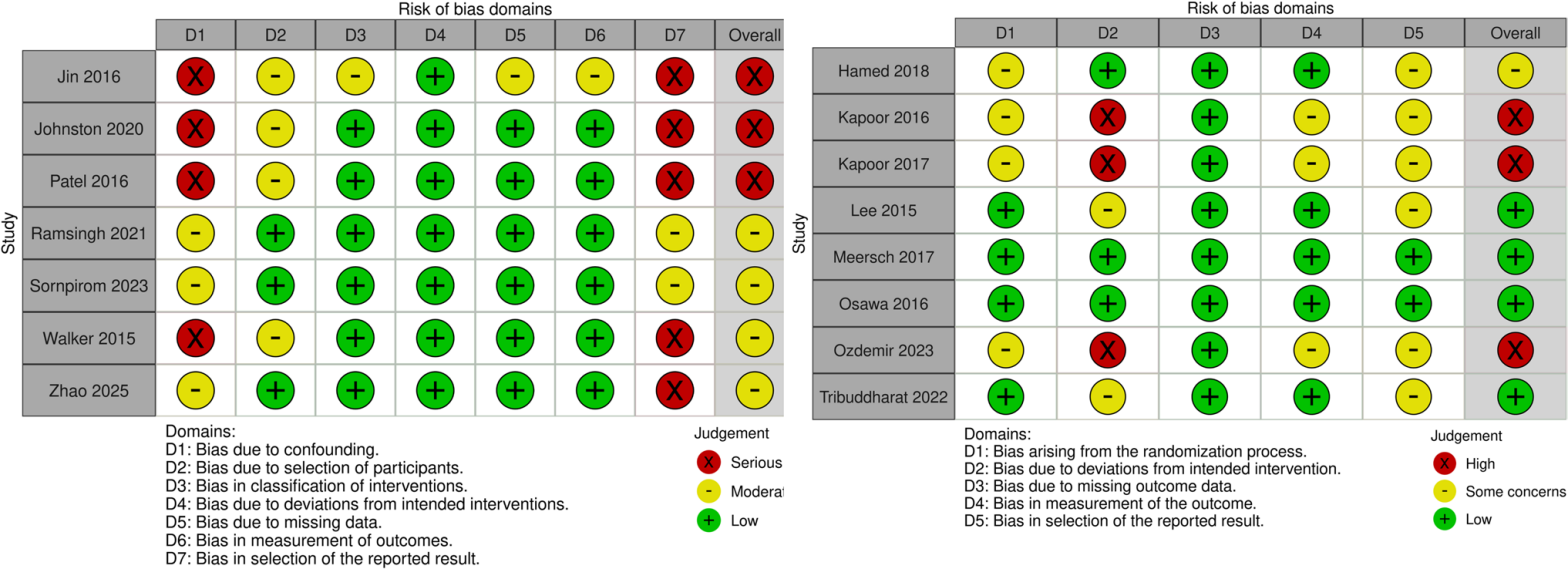
Risk of Bias assessment of the included studies using RoB 2 tool for randomized controlled trials and ROBINS-l for observational studies

Figure 2 displays the risk of bias assessment for all included studies. Among the RCTs, three were rated as having high risk of bias, primarily due to incomplete blinding and the absence of pre-specified analysis plans. In the observational studies, three were judged to be at serious risk of bias and four at moderate risk, mainly due to confounding, non-randomized allocation, and lack of protocol registration. Only four RCTs met the criteria for low risk of bias across all domains. A detailed assessment of each study across the respective domains is provided in the Supplementary Material.

## Hemodynamic monitoring strategies

### Invasive techniques

Among studies evaluating invasive hemodynamic monitoring strategies, the PAC remains the most widely utilized device for high-risk cardiac surgical patients. Five studies [14–18] employed PAC either as the primary monitoring modality or as a comparator to alternative techniques. Two randomized trials [14, 15] directly compared PAC to less invasive technologies—Vigileo-FloTrac and NICOM, respectively—in patients undergoing on-pump CABG and valve surgery with atrial fibrillation. In both studies, PAC served as the reference method for GDT, guided by traditional variables such as pulmonary capillary wedge pressure (PCWP) and CI, while the comparator devices relied on dynamic or noninvasive metrics, including SVV and stroke volume response to passive leg raising (PLR).

[18] utilized PAC in a cohort undergoing mixed cardiac surgeries involving CPB, including CABG and valve procedures. Their protocol incorporated mixed venous oxygen saturation (SvO₂) and lactate levels alongside conventional PAC-derived parameters. Similarly, [14] implemented PAC-guided GDT in patients undergoing CABG, valve, or combined procedures, using SvO₂ as a central marker for titrating inotropic support and determining transfusion thresholds. [17] adopted a multimodal strategy that included PAC as part of a comprehensive hemodynamic assessment following cardiac surgery, combining it with pulse pressure variation (PPV), PLR, and bedside echocardiography to evaluate fluid responsiveness.

These studies predominantly targeted moderate to high-risk patients undergoing complex cardiac procedures requiring CPB, where PAC use was justified by its capacity to provide detailed pressure and output data. However, considerable heterogeneity existed regarding patient profiles and the integration of PAC with other monitoring technologies, underscoring its evolving role in contemporary cardiac anesthesiology. Notably, PAC did not demonstrate superiority over less invasive approaches. In the randomized studies by [14, 15], the alternative technologies yielded comparable or even superior outcomes regarding fluid management and vasopressor requirements. In the remaining studies, PAC was either not directly compared or used as part of multi-modal strategies, limiting the ability to isolate its individual impact.

### Minimally invasive devices

Minimally invasive monitoring strategies were featured in nine of the included studies, with the FloTrac/EV1000® system emerging as the most frequently used platform, appearing in seven studies [11–14, 19–21]. These systems derive key hemodynamic variables—such as CO, SVV, and systemic vascular resistance index (SVRI)—from arterial waveform analysis. In addition to FloTrac/EV1000, other platforms included LiDCOrap-id® and esophageal Doppler, which were sometimes used in conjunction with EV1000®.

Most studies applied these technologies in patients undergoing either on-pump or off-pump CABG, although the specific clinical contexts varied. Two studies [19, 22] assessed high-risk patients undergoing on-pump CABG using FloTrac alongside continuous central venous oxygen saturation (ScvO₂) monitoring. [11] extended the application to off-pump CABG, using a combination of EV1000, FloTrac, and PreSep sensors. Another study [12] focused exclusively on off-pump CABG in a younger, lower-risk cohort with fewer comorbidities. [21] and [13] included broader cardiac surgical populations but still centered their protocols on EV1000-guided optimization during and after separation from CPB. [20] described a quality improvement initiative in elective cardiac surgery patients managed with CPB, utilizing EV1000 in conjunction with central venous pressure monitoring and transesophageal echocardiography—without routine PAC placement.

Compared with PAC-based protocols, which were typically reserved for high-risk patients with complex surgical needs or compromised ventricular function, minimally invasive platforms were more commonly employed in elective, lower- to moderate-risk surgical populations. While some studies [16, 18] implemented PAC in patients with reduced ejection fraction or undergoing combined CABG and valve procedures, several studies using FloTrac/EV1000 specifically targeted off-pump CABG cases [11, 12]. In this setting, continuous hemodynamic data are beneficial, yet there is a clear priority to reduce procedural invasiveness. Similarly, [20] reported using EV1000 protocols in routine elective cases without PAC placement, reflecting a broader trend toward using less invasive monitoring in standard perioperative care. These findings highlight how device selection often reflects not only institutional preferences but also a risk-stratified approach to monitoring. PAC remains reserved for hemodynamically fragile patients, whereas minimally invasive systems are increasingly used to enhance precision in stable patients—without introducing additional procedural risk.

### Non-invasive monitoring

Non-invasive hemodynamic monitoring was employed in two studies [15, 23], each utilizing distinct flow-based technologies tailored to specific surgical populations. [15] used the NICOM™ bioreactance-based system (Cheetah Medical, USA) to guide GDT in patients with atrial fibrillation undergoing valvular heart surgery [15]. A key advantage of bioreactance is its independence from cardiac rhythm regularity and mechanical ventilation, two factors that limit the reliability of arterial waveform-based systems [15]. This made NICOM particularly appropriate for their cohort, which had a high prevalence of rhythm disturbances.

[23] implemented an esophageal Doppler-based device (CardioQ-ODM +, Deltex Medical) in patients undergoing elective CABG or valve surgery under CPB [23]. While technically semi-invasive due to esophageal probe placement, the modality is considered non-invasive in the context of avoiding vascular catheterization. This Doppler-based platform provided continuous measurements of stroke volume index (SVI), CI, peak velocity (PV), and corrected flow time (FTc), and was used within a structured GDT algorithm based on SVI thresholds and dynamic responses to fluid infusion. Compared with PAC-based approaches, these non-invasive strategies were applied in more stable, elective surgical populations with preserved or mildly impaired ventricular function and without severe multivalvular pathology or significant comorbidities. Their use reflects a careful balance between procedural simplicity and the need for reliable, real-time hemodynamic data. A concise overview of the hemodynamic monitoring technologies identified across the included studies is presented in Table 2.

**Table 2:** Hemodynamic monitoring technologies used across included studies

Hemodynamic Monitoring Device	Measurement Principle	Invasiveness	Parameters	Indications	Key Limitations
Pulmonary Artery Catheter	Thermodilution via pulmonary artery catheterization	Invasive	CO, CI, SvO₂, PCWP, PAP, CVP	High-risk/complex cardiac surgery; impaired ventricular function; combined procedures	Procedural risk (arrhythmia, infection); limited outcome benefit; operator dependent
Pulse Contour Analysis (FloTrac/EV1000, LiDCOrapid)	Continuous CO estimation from arterial pressure waveform analysis	Minimally invasive	CO, CI, SVV, SVRI	On- or off-pump CABG; valve surgery;	Affected by arrhythmia, vasoplegia, and low SVR; uncalibrated
Esophageal Doppler (CardioQ-ODM +)	Blood-flow velocity in descending aorta via esophageal probe	Semi-invasive	CO, CI, PV, FTc, SVRI	Elective cardiac surgery; lower-risk or off-pump cases;	Requires probe positioning; contraindicated in esophageal pathology; less reliable in aortic pathology
Bioreactance (NICOM™)	Phase-shift analysis of thoracic electrical currents	Non-invasive	CO, CI, SV, ΔSVI (PLR)	Valvular surgery with atrial fibrillation or spontaneous breathing; postoperative monitoring	Lower accuracy in extreme edema or rapid hemodynamic changes
Transpulmonary Thermodilution (PiCCO™/VolumeView™)	Intermittent transpulmonary thermodilution combined with continuous arterial pulse contour analysis	Invasive	CO, CI, GEDV, GEF, EVLW, SVV, SVRI	High-risk cardiac surgery and ICU patients requiring detailed volumetric preload assessment and lung water monitoring; complex perioperative hemodynamic management	Requires dedicated central venous and arterial catheters and calibration; increased invasiveness and cost; does not directly assess right-sided pressures, RV function, or pulmonary vascular dynamics

Transpulmonary thermodilution systems (e.g., PiCCO™, VolumeView™) are included as widely used perioperative and ICU monitoring tools, although they were not used as primary GDT modalities in the trials summarized in this review. CABG, coronary artery bypass grafting; CI, cardiac index; CO, cardiac output; CVP, central venous pressure; EVLW, extravascular lung water; FTc, corrected flow time; GDT, goal-directed therapy; GEDV, global end-diastolic volume; GEF, global ejection fraction; PAP, pulmonary artery pressure; PCWP, pulmonary capillary wedge pressure; PV, peak velocity; SV, stroke volume; SVR(I), systemic vascular resistance (index); SVV, stroke volume variation

### Clinical implications

**Fig. 3:**
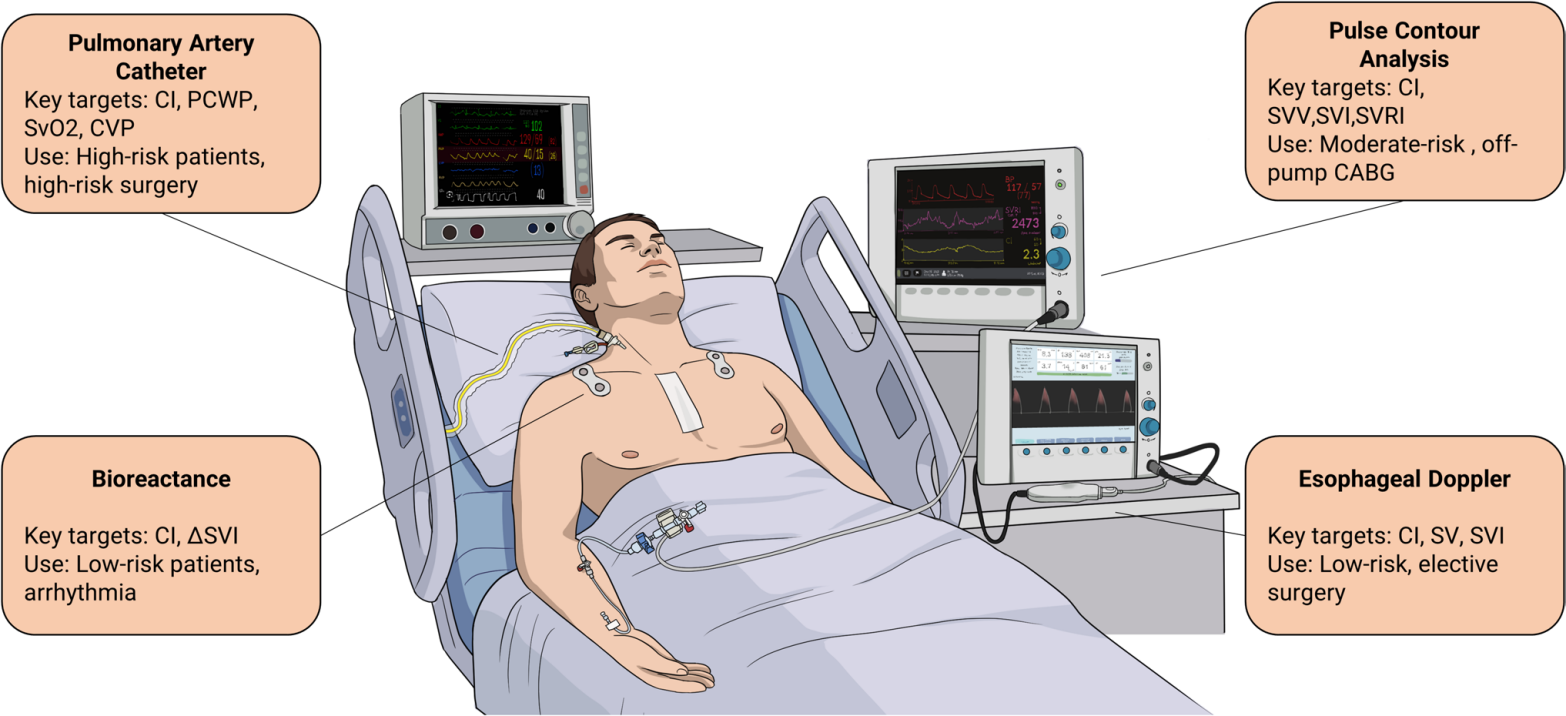
Hemodynamic monitoring tools used in cardiac surgery over the past 10 years. CABG: coronary artery bypass grafting; CI: cardiac index; CVP: central venous pressure; ΔSVI: change in stroke volume; PCWP: pulmonary capillary wedge pressure; SV: stroke volume; SVI: stroke volume index; SVV: stroke volume variation; SvO2: mixed venous oxygen saturation

Hemodynamic monitoring in cardiac surgery increasingly reflects a risk-adapted, patient-centered approach. Over the past 15 years, the range of available monitoring tools has expanded significantly, with minimally invasive platforms—particularly FloTrac/EV1000—emerging as the most frequently used technologies across a wide spectrum of studies (Fig. 3). PAC remains reserved for high-risk patients undergoing complex procedures, where detailed pressure and output metrics can support nuanced management decisions. In contrast, minimally invasive platforms are now commonly employed in moderate-risk populations, offering continuous hemodynamic monitoring with lower procedural risk. Non-invasive methods, including bioreactance and esophageal Doppler technologies, have demonstrated feasibility and clinical utility in selected low-risk, elective surgical settings. Their integration into perioperative protocols highlights an evolving preference for tailoring monitoring strategies to patient risk and surgical complexity, aiming to optimize outcomes while minimizing invasiveness and resource burden.

## Hemodynamic targets

### Cardiac index

CI (expressed in L/min/m^2^) was the most consistently targeted hemodynamic variable, included in 12 of the 15 reviewed protocols across both invasive and non-invasive strategies [11–23]. Most studies used a lower threshold between 2.0 and 2.5 L/min/m^2^ to initiate fluid therapy or inotropic support. However, three studies [11, 19, 22] used higher targets, ranging from ≥ 2.5 to ≥ 4.2 L/min/m^2^, particularly in off-pump or high-risk patients.

Higher targets were often associated with more aggressive fluid administration in the early postoperative period, sometimes without a corresponding increase in inotropic use, indicating that volume expansion alone was sufficient to meet perfusion goals. In studies by [11, 19], CI thresholds were part of a comprehensive GDT protocol that also tracked ScvO₂, DO₂I, and SVRI. Failure to achieve CI goals despite fluid resuscitation triggered early initiation of inotropes, typically dobutamine.

These findings suggest that while no universal CI threshold has been established, values are frequently adapted to patient acuity and surgical context. Lower targets were generally sufficient in stable, elective patients, while higher thresholds were pursued in cases requiring enhanced cardiac output or oxygen delivery.

### Fluid responsiveness

Assessment of fluid responsiveness was a fundamental element across most GDT protocols. There was a notable shift away from static preload measurements, such as central venous pressure (CVP) and PCWP, toward dynamic indicators like SVV and stroke volume response to PLR.SVV was the most frequently used parameter, reported in at least eight studies, particularly those employing the FloTrac/EV1000 platform [11–14, 19, 21]. Thresholds typically ranged from > 10% to > 13%, guiding volume administration within stepwise resuscitation protocols alongside CI or DO₂I target. These thresholds align with prior validation studies in mechanically ventilated patients with regular cardiac rhythm [24–26].

In addition to SVV, several studies used changes in SVI or CI following PLR or fluid bolus to assess fluid responsiveness [11, 15, 19, 23]. In the study by [15], a NICOM-derived ΔSVI ≥ 10% after PLR was used as a non-invasive surrogate for fluid responsiveness in postoperative patients with atrial fibrillation. This approach was designed to overcome the limitations of SVV in patients with arrhythmias or spontaneous respiratory activity. [16] assessed fluid responsiveness by evaluating CI before and after a 5 mL/kg bolus, considering a ΔCI > 10% as indicative of positive response. This was then used to determine further volume administration or escalation to vasoactive therapy. While some PAC-based studies reported CVP and PCWP values, these were not routinely used to guide fluid management decisions. Instead, they served as contextual or safety parameters. This further reflects the widespread shift toward physiology-based, dynamic indices to guide fluid therapy.

### Mean arterial pressure

MAP was a consistently targeted endpoint, though specific thresholds and therapeutic responses varied. Most protocols aimed to maintain MAP between 65 and 90 mmHg, using it in conjunction with CI or SVV to guide vasopressor and fluid strategies. In several studies [11, 15, 16, 27], MAP targets of 65–75 mmHg were employed, with vasoactive agents such as norepinephrine, phenylephrine, or vasopressin titrated based on CI and fluid responsiveness. [19] used a higher MAP target of 90–105 mmHg, especially in patients with vasoplegia or low SVRI after CPB, often requiring both vasopressors and inotropes. 

[13] applied a tiered protocol in which MAP guided therapy selection: phenylephrine was used when MAP was ≤ 65 mmHg with preserved CI, whereas dobutamine was chosen when MAP and CI were both low. This approach allowed MAP to serve both as a perfusion target and a decision point in pharmacologic escalation. Other studies [12, 23] incorporated SVRI into the hemodynamic decision-making process, emphasizing the interplay between perfusion pressure, vascular resistance, and CO. In these cases, dual-agent strategies (e.g., norepinephrine plus nitroglycerin or dobutamine) were employed to optimize hemodynamic balance.

Some protocols linked MAP targets to organ-specific endpoints. In two studies [27, 28], MAP ≥ 65 mmHg was incorporated into kidney protection strategies as part of multi-modal GDT protocols. Additionally, [20] adopted an individualized approach without predefined numerical targets, instead relying on intraoperative physiology, cerebral oximetry, and multidisciplinary consultation to determine appropriate hemodynamic goals. This strategy was associated with reduced inotropic support in the post-implementation cohort compared to historical controls (median reduction: –95.6 mL; p < 0.01).

In summary, these findings demonstrate that MAP is best utilized as a contextual and complementary endpoint within broader hemodynamic strategies, rather than as an isolated target. Its role in guiding vasopressor use is increasingly nuanced, shaped by concurrent flow parameters and patient-specific physiologic responses.

### Other hemodynamic and perfusion targets

In addition to conventional flow and pressure-based endpoints, several studies incorporated advanced hemodynamic and biochemical markers to further personalize perioperative management. ScvO₂ and SvO₂ were targeted in four studies [11, 17, 19, 27], with threshold goals typically set at ScvO₂ ≥ 70% and SvO₂ > 60%, particularly in post- CPB settings and in protocols aimed at acute kidney injury (AKI) prevention. Several protocols also included DO₂I targets, generally between 450 and 600 mL/min/m^2^, used alongside SVRI to guide decisions regarding fluid resuscitation versus initiation of inotropes or vasopressors [11, 12, 23]. Biochemical markers such as lactate and Cystatin-C were integrated into GDT protocols in three studies [23, 25, 27] as indicators of tissue perfusion adequacy and early renal dysfunction. These parameters were not used in isolation but rather embedded in multimodal decision-making frameworks to support timely recognition of hypoperfusion and early therapeutic intervention.

### Clinical implications

GDT protocols in cardiac surgery most frequently centered on optimization of CI and dynamic fluid responsiveness parameters, with MAP serving as a complementary perfusion target. The addition of adjunctive markers—including ScvO₂, DO₂I, lactate, and renal biomarkers—allowed for more individualized, physiology-driven strategies tailored to tissue oxygen delivery and end-organ protection. This layered approach reflects a growing trend toward precision hemodynamic management in complex surgical populations.

## Impact of goal-directed therapy on outcomes

### Respiratory support and ventilatory weaning

Multiple studies investigated the impact of GDT on respiratory recovery, particularly the duration of mechanical ventilation and time to extubation. Five studies reported shorter ventilation times or earlier extubation in patients managed with GDT compared with standard care [11, 13, 14, 18, 19]. These benefits were generally attributed to optimized volume status and preservation of CO, reducing pulmonary congestion and facilitating more efficient gas exchange. [16] reported greater intraoperative colloid use in the GDT group, while others such as [11], [13], and [18] implemented restrictive fluid strategies, leading to lower net fluid balances. One study [16], however, found no difference in ventilation duration and reported higher reintubation rates in the GDT group, potentially reflecting fluid overload due to strict protocol adherence despite adequate perfusion. The most effective protocols appeared to integrate real-time flow-based parameters, particularly CI and SVV, to balance perfusion needs with fluid restriction. [22], although not focused on respiratory outcomes, also observed earlier extubation trends in the GDT group.

### Renal protection and acute kidney injury

Several studies addressed the role of GDT in preserving renal function in the perioperative setting. One study [28] utilized a NephroCheck®-based strategy, triggering early hemodynamic optimization in response to elevated urinary TIMP-2·IGFBP7 levels. This biomarker-driven approach led to a significant reduction in postoperative AKI incidence. Other protocols incorporated ScvO₂ and lactate [27], or combined Cystatin-C monitoring with flow-based indices from minimally invasive platforms [23]. [17] also demonstrated reduced AKI rates after implementing a multimodal perfusion optimization bundle. Collectively, these findings underscore the value of integrating tissue oxygenation markers and renal stress indicators into GDT protocols to enhance early detection and mitigate organ injury.

### ICU and hospital length of stay

Five studies reported a significant reduction in ICU length of stay (LOS) with GDT implementation [11, 12, 19, 21, 22], and three also documented reductions in total hospital LOS [19, 21, 22]. These protocols commonly included structured algorithms guided by CI and SVV. In contrast, three studies [15, 16, 23] found no significant difference in LOS. For example, higher reintubation rates in [16] may have offset potential gains in ICU efficiency, while [15] attributed their neutral results to the use of non-invasive monitoring in patients with arrhythmia, limiting volume optimization precision. Despite variability in institutional discharge practices, these results suggest that well-structured GDT protocols can contribute to more efficient recovery trajectories and resource utilization, especially in high-risk surgical populations.

### Morbidity and mortality

GDT was associated with a reduction in selected postoperative complications across several studies. [22] reported lower infection rates and reduced incidence of low cardiac output syndrome. [21] noted fewer surgical site infections, and [23] and [15] observed decreased transfusion requirements, aligning with the use of more restrictive fluid strategies in GDT arms. Notably, these morbidity improvements were primarily seen in studies utilizing minimally invasive or non-invasive monitoring platforms. PAC-based studies did not consistently report or analyze patient-centered morbidity outcomes, potentially limiting their observed benefit.

Mortality was formally assessed in only one study [22], which demonstrated a significant reduction in 30-day mortality in the GDT group. Most other trials did not include mortality as a primary endpoint, limiting the ability to draw firm conclusions regarding survival benefit.

### Clinical implications

**Fig. 4:**
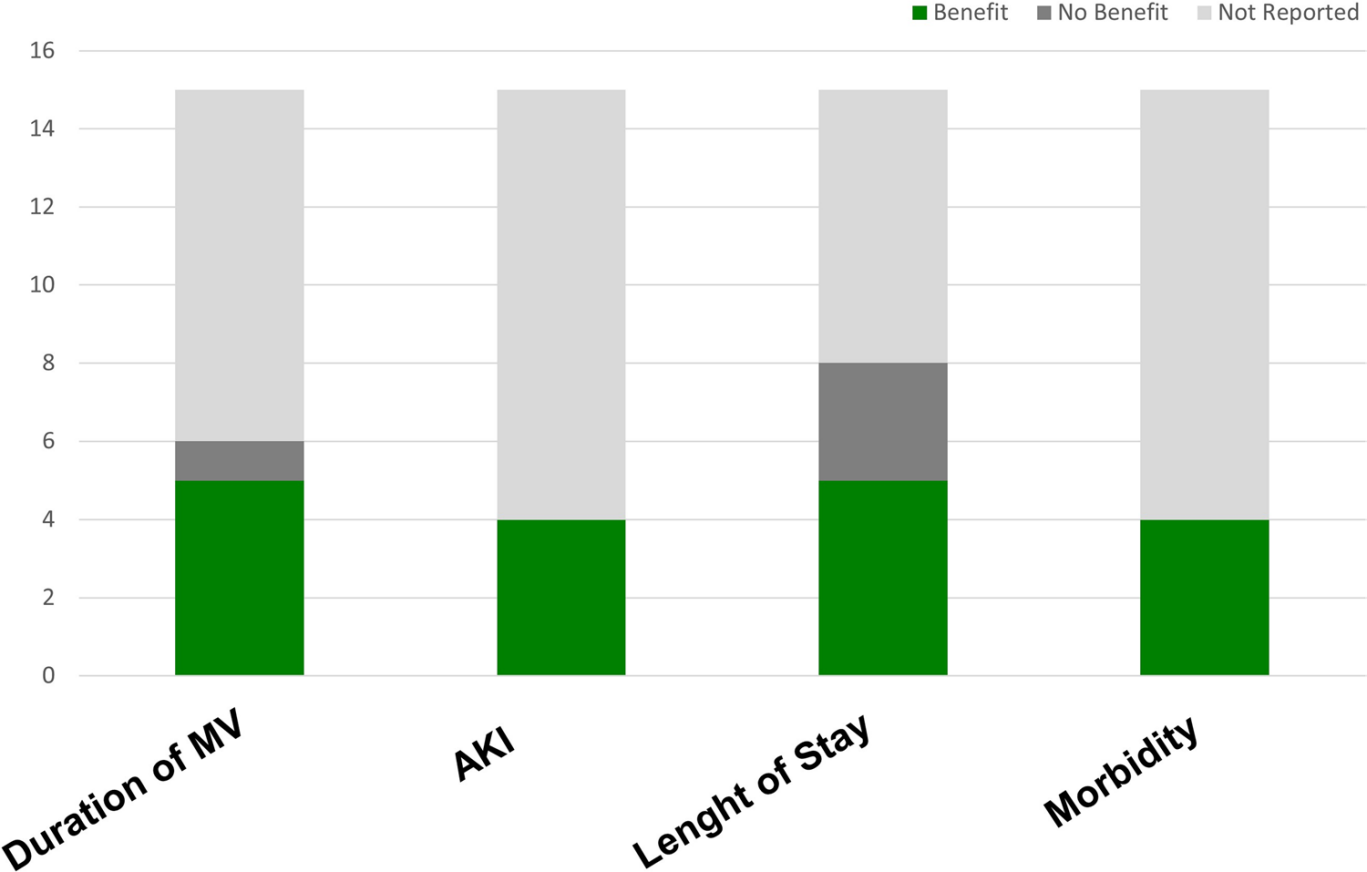
Impact of hemodynamic monitoring strategies on outcomes in cardiac surgery: number of studies reporting benefit (green), no benefit (gray), or not reported (light gray). AKI: acute kidney injury; MV: mechanical ventilation

While the current evidence supports improved postoperative recovery and complication reduction with GDT (Fig. 4) —particularly when minimally invasive monitoring is employed—its effect on mortality remains unclear and merits further investigation through adequately powered, outcome-focused trials.

## Discussion and future perspectives

**Fig. 5:**
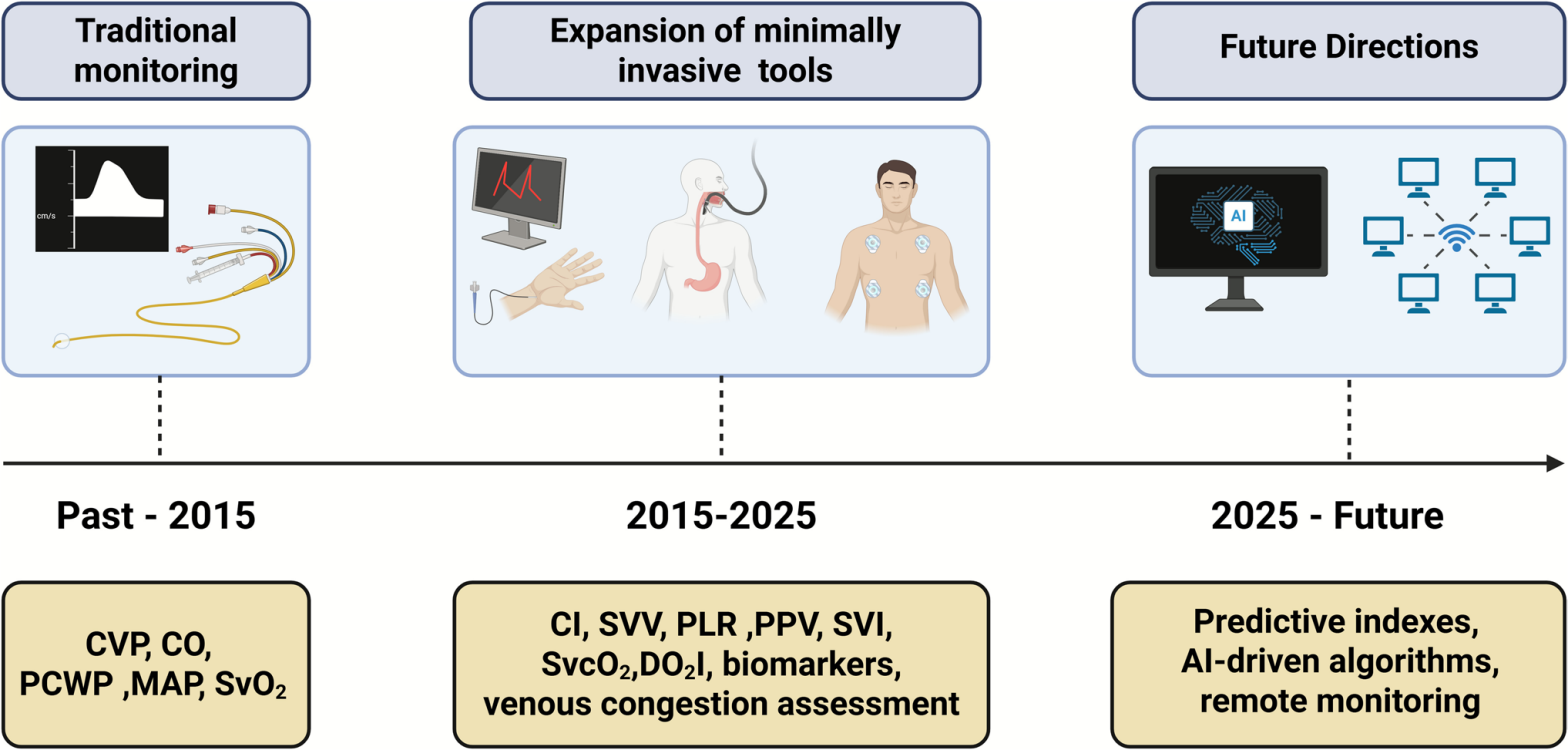
Timeline of hemodynamic monitoring strategies and targets in cardiac surgery, highlighting the shift from invasive tools to minimally invasive, non-invasive, and AI-driven approaches. AI: artificial intelligence; CI: cardiac index; CO: cardiac output; CVP: central venous pressure; DO2I: oxygen delivery indices; MAP: mean arterial pressure; PCWP: pulmonary capillary wedge pressure; PPV: pulse pressure variation; SV: stroke volume; SVI: stroke volume index; SVV: stroke volume variation; SvO2: mixed venous oxygen saturation

Despite the increased adoption and evolution of hemodynamic monitoring technologies in cardiac surgery, a consistent and evidence-based framework for GDT remains lacking (Fig. 5). The studies included in this review reveal substantial variability in monitoring strategies, target thresholds, and outcome reporting. While CI, SVV, and MAP were commonly used targets, thresholds varied significantly—ranging from permissive to aggressive perfusion strategies—particularly in off-pump or high-risk procedures.

Another key limitation is the predominance of surrogate endpoints such as AKI, ventilation time, or ICU stay. Only one study formally assessed 30-day mortality and demonstrated a benefit [22]. The lack of uniform outcome definitions and underreporting of complications like infection, bleeding, or myocardial injury hinder cross-study comparisons and limit the generalizability of findings.

Moreover, the overall methodological quality of the included studies was limited. Three of the seven RCTs were rated as high risk of bias, primarily due to lack of blinding and absence of pre-specified analysis plans—limitations that are, to some extent, inherent to the nature of hemodynamic monitoring interventions, which often preclude full blinding. Most observational studies were judged to have moderate to serious risk of bias, mainly due to confounding and non-randomized allocation. Additionally, the absence of protocol registration and clearly predefined outcomes was common across study designs, raising concerns about selective reporting. These limitations underscore the need for high-quality, adequately powered randomized trials using standardized definitions and clinically meaningful endpoints.

Implementation science is another major gap. Although some studies reported improvements in ICU efficiency, few addressed the cost-effectiveness of GDT protocols or the logistical challenges of integrating advanced monitoring technologies into routine perioperative care—especially in resource-limited environments. Heterogeneity in institutional practice patterns, protocol adherence, and clinician training further complicates real-world application.

Emerging technologies such as artificial intelligence (AI)–driven algorithms and implantable remote monitoring systems offer exciting opportunities. Early data suggest that AI can predict hypotension or fluid responsiveness from continuous waveform data [29–31]. The recently published HYPE-2 trial demonstrated that an AI-derived Hypotension Prediction Index significantly reduced both the depth and duration of intraoperative and postoperative hypotension in elective cardiac surgery patients, supporting its potential integration into hemodynamic protocols [32]. In parallel, implantable systems like the Cordella™ and CardioMEMS™ platform show potential for post-discharge monitoring of pulmonary artery pressures in chronic heart failure [33, 34], though their role in acute surgical care remains speculative.

Additionally, an increasingly recognized consideration in hemodynamic management is not only the evaluation of fluid responsiveness or perfusion-based hemodynamic targets, but also fluid tolerance, particularly in the context of venous congestion. Traditional preload-guided resuscitation may overlook the adverse effects of elevated venous pressures on organ perfusion. In recent years, the Venous Excess Ultrasound Score (VExUS) has gained attention as a bedside tool to assess systemic congestion by integrating Doppler-based measurements of hepatic, portal, and renal vein flow patterns with inferior vena cava (IVC) assessment [35, 36]. This score was originally developed and validated in a cohort of post-cardiac surgery patients, demonstrating a strong association between elevated venous congestion and the subsequent development of AKI. This relationship was further supported by a recent meta-analysis, which reported the strongest predictive value for AKI in the cardiac surgery subgroup compared to other critically ill populations [37]. These findings suggest that evaluating venous congestion—using tools like VExUS—could potentially complement existing perioperative hemodynamic monitoring strategies. Incorporating venous congestion assessment into perioperative protocols may help identify patients at risk of fluid-related organ dysfunction despite appearing fluid responsive, thereby refining fluid administration strategies to better balance perfusion goals with the risk of congestion.

To advance the field, there is an urgent need for multicenter, pragmatic clinical trials that incorporate standardized endpoints, stratify patients by risk, and evaluate both clinical efficacy and economic feasibility. Adaptive trial designs may further support the development of tailored hemodynamic monitoring and GDT strategies across diverse surgical contexts—including off-pump CABG, valve surgery, and combined procedures.

## Conclusion

Hemodynamic monitoring is essential in cardiac surgery, guiding fluid management, vasoactive therapy, and complication prevention. This review explores the range of tools—from PAC to non-invasive systems—each suited to specific patient risks and surgical demands. Despite integration into GDT protocols, choosing the right modality remains a challenge due to variability in device accuracy, thresholds, and clinical outcomes. As Cleobulus of Lindos, one of the Seven Sages of ancient Greece, once stated: “Measure is best.” In cardiac surgery, where precision can mean the difference between recovery and complication, this ancient wisdom remains strikingly relevant.

## Supplementary Information

Below is the link to the electronic supplementary material.

Supplementary file1

## Data Availability

No new data were created or analyzed in this study. Data sharing is not applicable to this article.
