# Supplementary material for: Hemodynamic monitoring strategies in cardiac surgery: an update systematic review
Source: J Clin Monit Comput. 2026 Jan 31;40(1):7–20. doi: 10.1007/s10877-025-01407-7 (PMC12963186; doi:10.1007/s10877-025-01407-7)
Supplement: Supplementary file 1 — Supplementary file1 [file 10877_2025_1407_MOESM1_ESM.docx]

**SUPPLEMENTARY MATERIAL**

**Table of Contents**

**Supplemental Methods 1 – Detailed Search Strategy3**

**Supplemental Methods 2 – Risk of Bias – RoB24**

**Supplemental Methods 3 – Risk of Bias – ROBINS-l........................................................ ......12**

**PRISMA Checklist 19**

**Supplemental Methods 1 – Detailed Search Strategy**

| **Pubmed** | ("cardiac surgical procedures"[MeSH Terms] OR "cardiac surgery" OR "cardiothoracic surgery" OR "heart surgery" OR "valve surgery" OR ''coronary artery bypass grafting'' OR CABG)  AND  ("goal-directed therapy" OR "goal directed therapy" OR GDT OR "hemodynamic monitoring" OR "hemodynamic assessment" OR "invasive monitoring" OR "noninvasive monitoring")  AND  ("2015/01/01"[Date - Publication] : "2025/12/31"[Date - Publication]) |
| --- | --- |
| **Embase** | ('cardiac surgical procedure'/exp OR 'cardiac surgery' OR 'cardiothoracic surgery' OR 'heart surgery' OR 'valve surgery' OR 'coronary artery bypass grafting' OR CABG)  AND  ('goal directed therapy' OR GDT OR 'hemodynamic monitoring' OR 'hemodynamic assessment' OR 'invasive monitoring' OR 'noninvasive monitoring')  AND  [2015-2025]/py |
| **Cochrane Library** | ("cardiac surgery" OR "cardiothoracic surgery" OR "heart surgery" OR "valve surgery" OR "coronary artery bypass grafting" OR CABG)  AND  ("goal-directed therapy" OR "goal directed therapy" OR GDT OR "hemodynamic monitoring" OR "hemodynamic assessment" OR "invasive monitoring" OR "noninvasive monitoring") |
| **Scopus** | (TITLE-ABS-KEY("cardiac surgery" OR "cardiothoracic surgery" OR "heart surgery" OR "valve surgery" OR "coronary artery bypass grafting" OR CABG))  AND  (TITLE-ABS-KEY("goal-directed therapy" OR "goal directed therapy" OR GDT OR "hemodynamic monitoring" OR "hemodynamic assessment" OR "invasive monitoring" OR "noninvasive monitoring"))  AND  PUBYEAR > 2014 AND PUBYEAR < 2026 |

**Supplemental Methods 2 – Risk of Bias – RoB2**

| **Hamed 2018** |
| --- |
| Domain 1: Bias arising from the randomization processJudgment: Some concernsJustification: The paper mentions that patients were randomized using a computer-generated list, but there is no information about allocation concealment (e.g., sealed envelopes, third-party allocation). Without this, baseline differences might occur due to subversion.Domain 2: Bias due to deviations from intended interventionsJudgment: Low riskJustification: Interventions were delivered according to the protocol (Group P: PAC; Group F: FloTrac/Vigileo). There is no indication of non-adherence or deviations likely to affect the outcome. The study is not blinded, but the primary outcomes (fluid volumes, ventilation time) are objective.Domain 3: Bias due to missing outcome dataJudgment: Low riskJustification: All 60 randomized patients completed the trial and were analyzed. There is no report of dropouts or missing data.Domain 4: Bias in measurement of the outcomeJudgment: Low riskJustification: Outcomes (fluid volumes, respiratory support time, ICU/hospital stay) are objective and unlikely to be influenced by knowledge of intervention. Though not blinded, this domain does not raise concerns due to outcome nature.Domain 5: Bias in selection of the reported resultJudgment: Some concernsJustification: The protocol was not registered, and no pre-specified analysis plan is available. This opens the possibility of selective outcome reporting, especially since some outcomes (e.g., fluid balance, SVRI) are extensively reported while others like mortality are absent.  - **Overall Risk of Bias: Some concerns** |
| **Kapoor 2016** |
| **Domain 1: Bias arising from the randomization process**  **Judgment: Some concerns**  **The study used a “sealed envelope technique,” but did not clarify whether envelopes were opaque and sequentially numbered. Allocation concealment and random sequence generation are not fully described. No significant baseline imbalances were reported, but due to insufficient detail, this domain is rated as "some concerns."**  **Domain 2: Bias due to deviations from intended interventions**  **Judgment: High risk**  **The study was unblinded and required real-time management decisions based on hemodynamic data. The open-label design introduces a high risk of performance bias, particularly since treatment decisions (fluid and drug titration) could be influenced by knowledge of group assignment. Additionally, 10 patients were excluded post-randomization without an intention-to-treat analysis.**  **Domain 3: Bias due to missing outcome data**  **Judgment: Low risk**  **Ten patients were excluded before data analysis, but the reasons were provided (arrhythmias, use of intra-aortic balloon pump). The proportion and reasons for missing data appear balanced and unlikely to bias the results.**  **Domain 4: Bias in measurement of the outcome**  **Judgment: Some concerns**  **While many outcomes (e.g., ventilation time, ICU stay) were objective, others such as decisions to extubate or discharge may have been influenced by the lack of blinding. Outcome assessors were not blinded.**  **Domain 5: Bias in selection of the reported result**  **Judgment: Some concerns**  **There was no published protocol or statistical analysis plan. Selective outcome reporting cannot be excluded, particularly given the large number of reported outcomes.**  **Overall Risk of Bias: High**   - **The combination of unblinded intervention delivery, lack of intention-to-treat analysis, and insufficient information about allocation concealment leads to a high overall risk of bias.overall risk of bias is high.** |
| **Kapoor 2017** |
| **Domain 1: Bias arising from the randomization process**  **Judgment: Some concerns**  **The study states that patients were randomized using “sealed envelope technique,” but it does not clarify how the randomization sequence was generated or whether allocation concealment was implemented effectively. There were no baseline imbalances, but due to limited reporting, this domain is rated as "some concerns."**  **Domain 2: Bias due to deviations from intended interventions**  **Judgment: High risk**  **The trial was unblinded. Since treatment decisions were guided by invasive monitoring data and real-time adjustments (e.g., fluids, vasopressors), the lack of blinding could influence clinician behavior. Moreover, there is no mention of intention-to-treat analysis or any sensitivity analyses accounting for potential deviations. These factors raise a high risk of performance bias.**  **Domain 3: Bias due to missing outcome data**  **Judgment: Low risk**  **There is no indication of missing outcome data or loss to follow-up. All randomized patients appear to have been included in the analysis.**  **Domain 4: Bias in measurement of the outcome**  **Judgment: Some concerns**  **Outcomes such as lactate clearance and hemodynamic parameters are objectively measured. However, decisions involving extubation and discharge timing could be influenced by lack of blinding. Outcome assessors were likely not blinded.**  **Domain 5: Bias in selection of the reported result**  **Judgment: Some concerns**  **There is no trial registration or protocol available. Although multiple outcomes were reported in detail, selective reporting cannot be ruled out.**  **Overall Risk of Bias: High**   - **The open-label design, lack of protocol registration, and absence of intention-to-treat analysis result in a high overall risk of bias for this study.** |
| **Lee 2015** |
| **1. Bias arising from the randomization process**  **Judgment: Low risk**  **Justification: The study reports that randomization was performed using a computer-generated table, with allocation to PAC or NICOM groups. Baseline characteristics were mostly balanced, with the exception of a higher incidence of double valve surgery and stroke history in the NICOM group, which may reflect chance imbalances in small samples. However, no evidence suggests a failure in the randomization process itself.**  **2. Bias due to deviations from intended interventions**  **Judgment: Some concerns**  **Justification: The study was unblinded to caregivers due to the nature of the interventions (invasive vs. non-invasive monitoring). This lack of blinding could have influenced co-interventions, although the authors describe standardized GDT protocols. Nevertheless, without blinding, there's a possibility that decisions were unconsciously influenced by knowledge of group allocation.**  **3. Bias due to missing outcome data**  **Judgment: Low risk**  **Justification: There is no mention of missing data for primary or secondary outcomes. All randomized patients (n=58 after exclusions) seem to have been included in the analysis, and reasons for exclusion (conversion to sinus rhythm) are clearly described and appear unrelated to outcomes.**  **4. Bias in measurement of the outcome**  **Judgment: Low risk**  **Justification: Primary and secondary outcomes (e.g., hospital length of stay, vasopressor use, ventilator days) are objective and unlikely to be affected by knowledge of intervention allocation. Outcomes were measured consistently between groups.**  **5. Bias in selection of the reported result**  **Judgment: Some concerns**  **Justification: The trial is not registered and no pre-specified protocol is mentioned. Therefore, there is some concern regarding selective reporting—especially for secondary outcomes. However, all major expected outcomes for a hemodynamic GDT trial were reported.**  **Overall Risk of Bias Judgment: Some concerns**   - **This is primarily due to the lack of blinding and the absence of a trial registration or protocol, which introduces uncertainty about selective reporting and deviation from intended interventions.** |
| **Meersch 2017** |
| **Domain 1: Bias arising from the randomization process**  **Judgment: Low risk**  **Justification: Patients were randomized using a computer-generated sequence in a 1:1 fashion, and allocation concealment was achieved with sealed opaque envelopes. Baseline characteristics were well-balanced, and the randomization process was clearly described.**  **Domain 2: Bias due to deviations from intended interventions**  **Judgment: Low risk**  **Justification: Although the study was unblinded due to the nature of the intervention (goal-directed therapy guided by real-time biomarkers), the treatment protocol was standardized and pre-specified. Co-interventions were similar across groups, and adherence to the assigned intervention appears to have been maintained. An intention-to-treat analysis was performed.**  **Domain 3: Bias due to missing outcome data**  **Judgment: Low risk**  **Justification: All patients were accounted for, and there were no exclusions post-randomization. Missing data were minimal and balanced, and unlikely to affect the outcomes.**  **Domain 4: Bias in measurement of the outcome**  **Judgment: Low risk**  **Justification: Primary outcome (incidence of acute kidney injury) was objectively assessed based on KDIGO criteria, using standardized serum creatinine and urine output measurements. Lack of blinding of outcome assessors is unlikely to have influenced the measurement.**  **Domain 5: Bias in selection of the reported result**  **Judgment: Low risk**  **Justification: The trial was registered at clinicaltrials.gov (NCT02162017), and all primary and secondary outcomes reported in the manuscript match the registered protocol. A pre-specified statistical analysis plan is mentioned.**  **Overall Risk of Bias: Low**  **This trial demonstrates robust methodology, adequate allocation concealment, protocol adherence, and objective outcome assessment, with minimal concern for bias.** |
| **Osawa 2016** |
| **Domain 1: Bias arising from the randomization process**  **Judgment: Low risk**  **Justification: Randomization was performed using a computer-generated block randomization list, and allocation was concealed using opaque, sealed envelopes. Baseline characteristics were well balanced, indicating that the randomization process was successful.**  **Domain 2: Bias due to deviations from intended interventions**  **Judgment: Low risk**  **Justification: The trial was double-blinded. Both patients and caregivers were unaware of the assigned intervention, minimizing the risk of performance bias. Adherence to the study drug was reported, and there were no deviations likely to affect outcomes. An intention-to-treat analysis was used.**  **Domain 3: Bias due to missing outcome data**  **Judgment: Low risk**  **Justification: There was minimal missing data. All randomized patients were accounted for in the primary outcome analysis. Reasons for any missing data were documented and did not differ between groups.**  **Domain 4: Bias in measurement of the outcome**  **Judgment: Low risk**  **Justification: The primary outcome (postoperative AKI) was measured using standardized KDIGO criteria. Blinded outcome assessment was performed, and the measurement methods were consistent across groups.**  **Domain 5: Bias in selection of the reported result**  **Judgment: Low risk**  **Justification: The study was registered (ClinicalTrials.gov: NCT01758655), and outcomes were reported as pre-specified. There is no evidence of selective reporting.**  **Overall Risk of Bias: Low**  **This is a well-conducted, double-blind RCT with proper randomization, allocation concealment, and pre-registration. All RoB 2 domains meet low-risk criteria.** |
| **Ozdemir 2023** |
| **Domain 1: Bias arising from the randomization process**  **Judgment: Some concerns**  **Justification: The study states that patients were “randomly divided into two groups,” but it does not describe the method of random sequence generation or whether allocation was concealed. This insufficient reporting raises some concerns, even though baseline characteristics were balanced.**  **Domain 2: Bias due to deviations from intended interventions**  **Judgment: High risk**  **Justification: The trial was unblinded, and interventions required clinical decision-making based on real-time Doppler monitoring in one group. Since the medical team was aware of group assignments and could adjust fluids, inotropes, or vasopressors accordingly, the lack of blinding increases the risk of performance bias. There is also no mention of intention-to-treat analysis.**  **Domain 3: Bias due to missing outcome data**  **Judgment: Low risk**  **Justification: There were no missing outcome data. All randomized patients were included in the final analysis.**  **Domain 4: Bias in measurement of the outcome**  **Judgment: Some concerns**  **Justification: Outcomes such as ventilation time, ICU stay, and lactate levels are objectively measured. However, the study lacks blinding of outcome assessors and includes some clinician-driven decisions, such as extubation timing, which may be influenced by knowledge of group allocation.**  **Domain 5: Bias in selection of the reported result**  **Judgment: Some concerns**  **Justification: There is no mention of trial registration or a pre-specified analysis plan. Selective reporting cannot be ruled out.**  **Overall Risk of Bias: High**  **This judgment is based on high risk due to deviations from intended interventions in an unblinded setting, and some concerns in other domains due to incomplete methodological reporting.** |
| **Tribuddharat 2022** |
| **Domain 1: Bias arising from the randomization process**  **Judgment: Low risk**  **Justification: Randomization was performed using a computer-generated list with block randomization. Allocation was concealed using sealed, opaque envelopes. Baseline characteristics were well balanced, suggesting successful randomization.**  **Domain 2: Bias due to deviations from intended interventions**  **Judgment: Some concerns**  **Justification: The trial was unblinded to the care team, and hemodynamic goals required individualized fluid and drug titration. Although the protocol was followed, the lack of blinding in an intervention-dependent trial raises concerns about potential performance bias. An intention-to-treat analysis was not explicitly stated.**  **Domain 3: Bias due to missing outcome data**  **Judgment: Low risk**  **Justification: All randomized patients completed the study and were included in the outcome analyses. There were no exclusions or losses to follow-up.**  **Domain 4: Bias in measurement of the outcome**  **Judgment: Low risk**  **Justification: Outcomes (e.g., fluid administered, lactate levels, postoperative complications) were objectively measured and unlikely to be influenced by lack of blinding. No indication that outcome assessors were biased.**  **Domain 5: Bias in selection of the reported result**  **Judgment: Some concerns**  **Justification: The study was not registered, and no pre-specified analysis plan was reported. Therefore, selective outcome reporting cannot be excluded.**  **Overall Risk of Bias: Some concerns**  **Despite a clearly defined protocol and complete data reporting, the lack of blinding and absence of trial registration introduce uncertainty in two key domains.** |

**Supplemental Methods 3 – Risk of Bias – ROBINS-l**

| **Jin 2016** |
| --- |
| **Domain 1: Bias due to confounding**  **Judgment: Serious**  **Justification: The authors state that the GDT group had more complex surgeries and longer bypass time. Although multivariable regression was used, residual confounding is likely due to the non-randomized design and baseline differences. Confounding by indication cannot be excluded.**  **Domain 2: Bias in selection of participants into the study**  **Judgment: Moderate**  **Justification: The study included consecutive patients undergoing CABG surgery, minimizing selection bias. However, the retrospective grouping based on intervention use introduces some risk that group allocation was influenced by unmeasured factors (e.g., clinician preference).**  **Domain 3: Bias in classification of interventions**  **Judgment: Moderate**  **Justification: Patients were classified based on whether they received GDT or conventional care, but the exact criteria for group assignment are not fully described. Misclassification is unlikely, but cannot be ruled out entirely.**  **Domain 4: Bias due to deviations from intended interventions**  **Judgment: Low**  **Justification: Given the retrospective design, there’s no indication that deviations from protocol occurred based on intervention knowledge. Outcomes were analyzed according to actual interventions received.**  **Domain 5: Bias due to missing data**  **Judgment: Low**  **Justification: The study included all patients with complete surgical records. There is no indication of significant missing outcome or covariate data.**  **Domain 6: Bias in measurement of outcomes**  **Judgment: Low**  **Justification: Most outcomes (e.g., ICU stay, fluid balance, postoperative complications) were objectively recorded in medical charts and not likely affected by knowledge of intervention status.**  **Domain 7: Bias in selection of the reported result**  **Judgment: Serious**  **Justification: The study was not registered, and there is no pre-specified protocol. Multiple outcomes are reported, and selective outcome reporting cannot be ruled out.**  **Overall Risk of Bias: Serious**  **This is primarily due to confounding (non-random allocation, group imbalances) and the absence of a prespecified analysis plan.** |
| **Johnston 2020** |
| **Domain 1: Bias due to confounding**  **Judgment: Serious**  **Justification: The GDT group had higher baseline risk: significantly more patients with diabetes and redo surgeries, and longer cardiopulmonary bypass times. Although the authors used regression modeling, they did not perform propensity matching or inverse probability weighting, and residual confounding likely persists.**  **Domain 2: Bias in selection of participants into the study**  **Judgment: Moderate**  **Justification: Patients were grouped based on whether they were managed before or after GDT protocol implementation. While all patients were included during a defined period, the grouping by time introduces potential temporal bias (e.g., co-interventions or institutional changes over time).**  **Domain 3: Bias in classification of interventions**  **Judgment: Low**  **Justification: Group classification is based on documented use of GDT protocol and timing of implementation. Misclassification is unlikely, as the protocol was institutional and prospectively applied after its introduction.**  **Domain 4: Bias due to deviations from intended interventions**  **Judgment: Low**  **Justification: Patients received usual care vs. care under a predefined GDT protocol. Since it’s a retrospective design, deviation from intended intervention is unlikely to be affected by knowledge of group assignment.**  **Domain 5: Bias due to missing data**  **Judgment: Low**  **Justification: No indication of substantial missing data. Outcome data were available for all included patients.**  **Domain 6: Bias in measurement of outcomes**  **Judgment: Low**  **Justification: Outcomes such as ICU stay, vasopressor use, AKI, and length of ventilation were objectively defined and extracted from electronic records. Unlikely to be influenced by knowledge of intervention.**  **Domain 7: Bias in selection of the reported result**  **Judgment: Serious**  **Justification: There was no pre-specified protocol or registration. The large number of reported outcomes increases the chance of selective reporting, especially for secondary endpoints.**  **Overall Risk of Bias: Serious**  **Primarily driven by confounding (baseline imbalance and lack of robust adjustment) and absence of protocol registration.** |
| **Patel 2016** |
| **Domain 1: Bias due to confounding**  **Judgment: Serious**  **Justification: There is no evidence of matching or statistical adjustment for confounding. The study does not present baseline characteristics or attempt to control for potential differences between groups. As this is a retrospective non-randomized study, confounding is a major concern.**  **Domain 2: Bias in selection of participants into the study**  **Judgment: Moderate**  **Justification: Patients were included based on the presence or absence of a GDT protocol during their care, but selection criteria are not clearly described. Group assignment may have been influenced by clinical judgment or availability of resources, leading to potential selection bias.**  **Domain 3: Bias in classification of interventions**  **Judgment: Low**  **Justification: Group classification (GDT vs. control) is based on medical record documentation of hemodynamic goals and protocol application. Classification appears to be accurate and reliable.**  **Domain 4: Bias due to deviations from intended interventions**  **Judgment: Low**  **Justification: As this was a retrospective analysis, deviations from intended interventions are unlikely to be influenced by the exposure status. The treatment protocol was standard once applied.**  **Domain 5: Bias due to missing data**  **Judgment: Low**  **Justification: The study does not report substantial missing data. Outcomes were analyzed for all patients included.**  **Domain 6: Bias in measurement of outcomes**  **Judgment: Low**  **Justification: Primary outcomes such as ICU stay, inotropic support, and complications were extracted from objective hospital records, and outcome assessors were likely blinded by design (retrospective nature).**  **Domain 7: Bias in selection of the reported result**  **Judgment: Serious**  **Justification: No registration or analysis plan was provided. Selective reporting is a concern, particularly since secondary outcomes and subgroup analyses are presented without prior definition.**  **Overall Risk of Bias: Serious**  **Driven by substantial concerns about confounding and the absence of a registered analysis plan or adjustment for baseline differences.** |
| **Ramsingh 2021** |
| **Domain 1: Bias due to confounding**  **Judgment: Moderate**  **Justification: The authors performed multivariable logistic regression adjusting for key variables (e.g., ASA classification, comorbidities). However, because allocation to GDT was not randomized, and residual confounding remains possible, risk is rated as moderate.**  **Domain 2: Bias in selection of participants into the study**  **Judgment: Low**  **Justification: All adult patients undergoing elective cardiac surgery at a single center were considered for inclusion over a defined time frame. Group assignment was based on implementation timing of a standardized protocol, which limits selection bias.**  **Domain 3: Bias in classification of interventions**  **Judgment: Low**  **Justification: Patients were clearly assigned to either GDT or standard care based on clinical pathway timing, and intervention classification is well documented and reliable.**  **Domain 4: Bias due to deviations from intended interventions**  **Judgment: Low**  **Justification: Since this was a pragmatic comparison of standard care vs. a new protocol, and no crossover or deviation is described, this domain is at low risk.**  **Domain 5: Bias due to missing data**  **Judgment: Low**  **Justification: The study accounted for all patients, and missing data were minimal or not reported. There’s no indication of differential loss between groups.**  **Domain 6: Bias in measurement of outcomes**  **Judgment: Low**  **Justification: Outcomes such as complications, ICU/hospital stay, and mortality were drawn from electronic records and assessed objectively.**  **Domain 7: Bias in selection of the reported result**  **Judgment: Moderate**  **Justification: The study was registered at ClinicalTrials.gov (NCT03432608), but there are some discrepancies between registered outcomes and reported endpoints (e.g., subgroup findings not pre-specified). No formal statistical analysis plan was uploaded.**  **Overall Risk of Bias: Moderate**  **This prospective cohort is methodologically strong with appropriate adjustment for confounders and complete outcome data. Remaining concerns stem primarily from non-random group allocation and some inconsistencies with pre-registration.** |
| **Sornpirom 2023** |
| **Domain 1: Bias due to confounding**  **Judgment: Moderate**  **Justification: The groups were matched by design on baseline characteristics (age, sex, LVEF, comorbidities), and no major imbalances were evident. However, the absence of randomization means residual confounding cannot be excluded, especially regarding perioperative management decisions.**  **Domain 2: Bias in selection of participants into the study**  **Judgment: Low**  **Justification: The study included consecutive eligible patients undergoing elective cardiac surgery during a clearly defined time frame, with predefined inclusion and exclusion criteria. Group assignment was based on Doppler availability and staff training, reducing risk of selection bias.**  **Domain 3: Bias in classification of interventions**  **Judgment: Low**  **Justification: Intervention classification (Doppler-guided GDT vs. standard care) was clear and based on real-time protocol use. There’s no evidence of misclassification.**  **Domain 4: Bias due to deviations from intended interventions**  **Judgment: Low**  **Justification: There were no deviations from the predefined treatment strategies reported. As the study was prospective, protocol adherence was closely monitored and documented.**  **Domain 5: Bias due to missing data**  **Judgment: Low**  **Justification: All 108 patients were analyzed. No missing primary or secondary outcome data were reported.**  **Domain 6: Bias in measurement of outcomes**  **Judgment: Low**  **Justification: Outcomes such as fluid balance, lactate clearance, ICU/hospital stay, and postoperative complications were objectively recorded. Blinding was unlikely but not essential due to objective measures.**  **Domain 7: Bias in selection of the reported result**  **Judgment: Serious**  **Justification: The study does not appear to have been registered, and no pre-specified analysis plan is available. Multiple outcomes were assessed, including several exploratory and subgroup findings, increasing the risk of selective reporting.**  **Overall Risk of Bias: Moderate**  **The study benefits from a prospective design, good baseline comparability, and complete outcome data, but non-randomized design and lack of prospective registration lead to moderate overall risk.** |
| **Walker 2015** |
| **Domain 1: Bias due to confounding**  **Judgment: Serious**  **Justification: The authors acknowledge that group assignment was determined by Doppler equipment availability and clinician preference, which introduces potential confounding by indication. There was no statistical adjustment for baseline differences, and important confounders such as intraoperative management strategies or fluid responsiveness were not controlled for.**  **Domain 2: Bias in selection of participants into the study**  **Judgment: Moderate**  **Justification: Although patients were prospectively enrolled and inclusion criteria were defined, the non-randomized allocation introduces moderate risk that certain patient characteristics influenced group assignment (e.g., high-risk patients being more or less likely to receive monitoring).**  **Domain 3: Bias in classification of interventions**  **Judgment: Low**  **Justification: The intervention (Doppler-guided GDT) was clearly defined and documented in the patient chart. No misclassification is likely.**  **Domain 4: Bias due to deviations from intended interventions**  **Judgment: Low**  **Justification: The care teams followed a defined protocol for Doppler use. There’s no indication that deviations occurred based on group assignment or awareness of the intervention.**  **Domain 5: Bias due to missing data**  **Judgment: Low**  **Justification: There were no losses to follow-up or exclusions after enrollment. All patients were included in outcome analyses.**  **Domain 6: Bias in measurement of outcomes**  **Judgment: Low**  **Justification: Primary outcomes such as length of stay, complications, and transfusion needs were objectively measured using chart data. Measurement bias is unlikely.**  **Domain 7: Bias in selection of the reported result**  **Judgment: Serious**  **Justification: The study was not registered, and the outcomes were not pre-specified. This raises the possibility of selective outcome reporting or post hoc analysis decisions.**  **Overall Risk of Bias: Serious**  **Despite a prospective design and complete data, lack of randomization, absence of adjustment for confounding, and no protocol registration result in serious risk of bias.** |
| **Zhao 2025** |
| **Domain 1: Bias due to confounding**  **Judgment: Moderate**  **Justification: The study presents balanced baseline characteristics and reports similar demographic and clinical features between groups. However, no formal statistical adjustment (e.g., multivariable regression or propensity score matching) was performed to address residual confounding.**  **Domain 2: Bias in selection of participants into the study**  **Judgment: Low**  **Justification: Patients were prospectively enrolled based on predefined inclusion/exclusion criteria. Allocation to groups appears to be based on time period or protocol implementation, minimizing selection bias.**  **Domain 3: Bias in classification of interventions**  **Judgment: Low**  **Justification: Intervention status (GDT vs. control) was clearly defined and prospectively recorded. Classification is accurate and well documented.**  **Domain 4: Bias due to deviations from intended interventions**  **Judgment: Low**  **Justification: There were no reports of crossover or protocol violations. Treatment followed standardized perioperative procedures according to the assigned protocol.**  **Domain 5: Bias due to missing data**  **Judgment: Low**  **Justification: The study reports complete data for all enrolled patients, and there is no indication of missing outcomes or loss to follow-up.**  **Domain 6: Bias in measurement of outcomes**  **Judgment: Low**  **Justification: All outcomes, such as fluid balance, hemodynamic stability, lactate levels, and adverse events, were objectively measured and based on electronic medical records or lab results.**  **Domain 7: Bias in selection of the reported result**  **Judgment: Serious**  **Justification: There was no pre-registration or published protocol. The study reports multiple outcomes, and it is unclear whether they were pre-specified. This raises concerns about selective reporting.**  **Overall Risk of Bias: Moderate**  **The study benefits from a prospective design and balanced groups, but the lack of statistical adjustment for confounders and absence of protocol registration justifies a moderate risk of bias overall.** |

| **Section and Topic** | **Item #** | **Checklist item** | **Location where item is reported** |
| --- | --- | --- | --- |
| **TITLE** | | |  |
| Title | 1 | Identify the report as a systematic review. | Pg 1 |
| **ABSTRACT** | | |  |
| Abstract | 2 | See the PRISMA 2020 for Abstracts checklist. | Pg 2 |
| ***INTRODUCTION*** | | |  |
| Rationale | 3 | Describe the rationale for the review in the context of existing knowledge. | Pg 3 |
| Objectives | 4 | Provide an explicit statement of the objective(s) or question(s) the review addresses. | Pg 3 |
| **METHODS** | | |  |
| Eligibility criteria | 5 | Specify the inclusion and exclusion criteria for the review and how studies were grouped for the syntheses. | Pg 4 |
| Information sources | 6 | Specify all databases, registers, websites, organisations, reference lists and other sources searched or consulted to identify studies. Specify the date when each source was last searched or consulted. | Pg 4 |
| Search strategy | 7 | Present the full search strategies for all databases, registers and websites, including any filters and limits used. | Pg 3 (Supplement) |
| Selection process | 8 | Specify the methods used to decide whether a study met the inclusion criteria of the review, including how many reviewers screened each record and each report retrieved, whether they worked independently, and if applicable, details of automation tools used in the process. | Pg 4 |
| Data collection process | 9 | Specify the methods used to collect data from reports, including how many reviewers collected data from each report, whether they worked independently, any processes for obtaining or confirming data from study investigators, and if applicable, details of automation tools used in the process. | Pg 4 |
| Data items | 10a | List and define all outcomes for which data were sought. Specify whether all results that were compatible with each outcome domain in each study were sought (e.g. for all measures, time points, analyses), and if not, the methods used to decide which results to collect. | Pg 4 |
|  | 10b | List and define all other variables for which data were sought (e.g. participant and intervention characteristics, funding sources). Describe any assumptions made about any missing or unclear information. | Pg 4 |
| Study risk of bias assessment | 11 | Specify the methods used to assess risk of bias in the included studies, including details of the tool(s) used, how many reviewers assessed each study and whether they worked independently, and if applicable, details of automation tools used in the process. | Pg 4 |
| Effect measures | 12 | Specify for each outcome the effect measure(s) (e.g. risk ratio, mean difference) used in the synthesis or presentation of results. | Pg 4 |
| Synthesis methods | 13a | Describe the processes used to decide which studies were eligible for each synthesis (e.g. tabulating the study intervention characteristics and comparing against the planned groups for each synthesis (item #5)). | Pg 3,4 |
|  | 13b | Describe any methods required to prepare the data for presentation or synthesis, such as handling of missing summary statistics, or data conversions. | Pg 3,4 |
|  | 13c | Describe any methods used to tabulate or visually display results of individual studies and syntheses. | Pg 3,4 |
|  | 13d | Describe any methods used to synthesize results and provide a rationale for the choice(s). If meta-analysis was performed, describe the model(s), method(s) to identify the presence and extent of statistical heterogeneity, and software package(s) used. | Pg 2-4 |
|  | 13e | Describe any methods used to explore possible causes of heterogeneity among study results (e.g. subgroup analysis, meta-regression). | - |
|  | 13f | Describe any sensitivity analyses conducted to assess robustness of the synthesized results. | - |
| Reporting bias assessment | 14 | Describe any methods used to assess risk of bias due to missing results in a synthesis (arising from reporting biases). | Pg 4,5, figure 2 |
| Certainty assessment | 15 | Describe any methods used to assess certainty (or confidence) in the body of evidence for an outcome. | - |
| **RESULTS** | | |  |
| Study selection | 16a | Describe the results of the search and selection process, from the number of records identified in the search to the number of studies included in the review, ideally using a flow diagram. | Pg 5 |
|  | 16b | Cite studies that might appear to meet the inclusion criteria, but which were excluded, and explain why they were excluded. | Figure 1 |
| Study characteristics | 17 | Cite each included study and present its characteristics. | Table 1 |
| Risk of bias in studies | 18 | Present assessments of risk of bias for each included study. | Pg 4(supplement)  Figure 2 |
| Results of individual studies | 19 | For all outcomes, present, for each study: (a) summary statistics for each group (where appropriate) and (b) an effect estimate and its precision (e.g. confidence/credible interval), ideally using structured tables or plots. | -- |
| Results of syntheses | 20a | For each synthesis, briefly summarise the characteristics and risk of bias among contributing studies. | Pg 4-18 (supplement) |
|  | 20b | Present results of all statistical syntheses conducted. If meta-analysis was done, present for each the summary estimate and its precision (e.g. confidence/credible interval) and measures of statistical heterogeneity. If comparing groups, describe the direction of the effect. | - |
|  | 20c | Present results of all investigations of possible causes of heterogeneity among study results. | - |
|  | 20d | Present results of all sensitivity analyses conducted to assess the robustness of the synthesized results. | - |
| Reporting biases | 21 | Present assessments of risk of bias due to missing results (arising from reporting biases) for each synthesis assessed. | - |
| Certainty of evidence | 22 | Present assessments of certainty (or confidence) in the body of evidence for each outcome assessed. | - |
| **DISCUSSION** | | |  |
| Discussion | 23a | Provide a general interpretation of the results in the context of other evidence. | Pg 6-18 |
|  | 23b | Discuss any limitations of the evidence included in the review. | Pg 18-19 |
|  | 23c | Discuss any limitations of the review processes used. | Pg 18-19 |
|  | 23d | Discuss implications of the results for practice, policy, and future research. | Pg 18-19 |
| **OTHER INFORMATION** | | |  |
| Registration and protocol | 24a | Provide registration information for the review, including register name and registration number, or state that the review was not registered. | Pg 2 |
|  | 24b | Indicate where the review protocol can be accessed, or state that a protocol was not prepared. | Pg 2 (PROSPERO) |
|  | 24c | Describe and explain any amendments to information provided at registration or in the protocol. | Pg 2 |
| Support | 25 | Describe sources of financial or non-financial support for the review, and the role of the funders or sponsors in the review. | Title page |
| Competing interests | 26 | Declare any competing interests of review authors. | Title page |
| Availability of data, code and other materials | 27 | Report which of the following are publicly available and where they can be found: template data collection forms; data extracted from included studies; data used for all analyses; analytic code; any other materials used in the review. | Supplementary material |
